# Effect of Daily Caper Fruit Pickle Consumption on Disease Regression in Patients with Non-Alcoholic Fatty Liver Disease: a Double-Blinded Randomized Clinical Trial

**DOI:** 10.15171/apb.2017.077

**Published:** 2017-12-31

**Authors:** Narjes Khavasi, Mohammad hosein Somi, Ebrahim Khadem, Elnaz Faramarzi, Mohammad Hossein Ayati, Seyyed Muhammad Bagher Fazljou, Mohammadali Torbati

**Affiliations:** ^1^Department of Traditional Medicine, Faculty of Traditional Medicine, Tabriz University of Medical Sciences, Tabriz, Iran.; ^2^Department of liver and Gastrointestinal Diseases Research Center, Tabriz University of Medical sciences, Tabriz, Iran.; ^3^Department of Traditional Medicine, School of Traditional Medicine, Tehran University of Medical Sciences, Tehran, Iran.; ^4^Department of Food Science and Technology, Faculty of nutrition, Tabriz University of Medical Sciences, Tabriz, Iran.

**Keywords:** Iranian traditional medicine, Non-alcoholic fatty liver, Caper fruit, Lipid profile

## Abstract

***Purpose:*** Despite numerous studies on the effects of complementary medicine, to our knowledge, there is no study on the effects of Capparis spinosa on disease regression in non-alcoholic fatty liver disease (NAFLD) patients. We compared the effects of caper fruit pickle consumption, as an Iranian traditional medicine product, on the anthropometric measures and biochemical parameters in different NAFLD patients.

***Methods:*** A 12-weeks randomized, controlled, double-blind trial was designed in 44 NAFLD patients randomly categorized for the control (n=22) or caper (n=22). The caper group received 40-50 gr of caper fruit pickles with meals daily. Before and after treatment, we assessed anthropometric measures, grade of fatty liver, serum lipoproteins and liver enzymes.

***Results:*** Weight and BMI were significantly decreased in the caper (p<0.001 and p<0.001) and control group (p=0.001 and p=0.001), respectively. Serum TG, TC and LDL.C just were significantly decreased in the control group (p=0.01, p<0.001 and p<0.001, respectively). Adjusted to the baseline measures, serum ALT and AST reduction were significantly higher in the caper than control group from baseline up to the end of the study (p<0.001 and p=0.02, respectively). After weeks 12, disease severity was significantly decreased in the caper group (p <0.001).

***Conclusion:*** Our results suggest that daily caper fruit pickle consumption for 12 weeks may be potentially effective on improving the biochemical parameters in NAFLD patients. Further, additional larger controlled trials are needed for the verification of these results.

## Introduction

Non-alcoholic fatty liver disease (NAFLD) is one of the increasing metabolic disorders which has a direct link with obesity, glucose intolerance, inflammatory pathways and dyslipidemia.^1^ Fatty liver disease refers to a wide range of liver damages, ranging from plain steatosis to steatohepatitis, advanced fibrosis, and finally cirrhosis.^2,3^ Drugs used to decrease NAFLD progression had inconclusive results.^4-9^ Because lifestyle modifications and drugs cannot be implemented effectively, new pharmacological and/or complementary foods are needed to be studied for reducing NAFLD progression. Currently, plants and/or functional foods have been noticed for the disease control or treatment due to the ease of access and in some cases, due to fewer side effects.^10^ Several traditional medicinal plants are used in different areas of the world to treat metabolic disorders.^11,12^ *Capparis spinosa* (caper) belongs to the family of Capparidaceae and is widely found in the southern area of Iran and the western or central regions of Asia.^13,14^ Caper’s root includes flavonoids, pectin, saponins, essential oils, tannins and particularly glycosinolate and glycosides as valuable biochemical compounds.^15,16^ Aqueous extract of C. *spinosa* showed blood glucose and lipid profile lowering effects in diabetic patients.^17,18^ Different parts of the plant, including fruits, leaves, seeds, etc., may have different effects due to various active ingredients. To our knowledge, human and animal studies have verified the positive effects of caper fruit on blood glucose in type 2 diabetic patients, and it has been used traditionally as an anti-hyperglycemic food by Iranian diabetic patients.^17-19^ Despite numerous studies about the beneficial effects of caper aqueous extract consumption on disease control in type 2 diabetic patients, no study, to our knowledge, has evaluated whether daily caper fruit consumption as a food additive can be effective in disease regression or not. Moreover, there is no human trial in this field.

The purpose of this study was to assess the effects of caper fruit consumption, as an Iranian Traditional Medicine product, change in biochemical parameters include serum lipids, liver enzymes and disease severity as primary outcome, and change in anthropometric or nutritional parameters as secondary outcome in NAFLD patients after 12 weeks. We hypothesized that daily caper fruit pickle consumption leads to the improvement in anthropometric measures, liver function tests, lipid profile and grade of fatty liver in patients with NAFLD.

## Materials and Methods

### Study participants and recruitment 

In total, 44 NAFLD patients were selected between March 2016 and April 2017 from among patients with NAFLD diagnosis who were attending the Metabolic Disease Research Center and Valie-Asr Hospital, Zanjan University of Medical Sciences, Zanjan, Iran. The inclusion criteria were as follows: patients aged 12-80 y, BMI (in kg/m^2^) of 25- 35, and willingness to consume caper fruit pickle as food additive. The exclusion criteria were any known allergies to caper, cigarette smoking, pregnancy or pregnancy planning in the next 6 months, breastfeeding, history of stroke, cirrhosis, viral hepatitis, liver obstructive diseases, heart disease or thyroid disorders, diabetes, dyslipidemia, intake of anti-diabetic or lipid lowering drugs, as well as anticoagulants, intake of medications that could affect body weight and/or energy expenditure, following vegetarian or weight-loss diets up to 2 months before the beginning of the study. Caper has interaction with coagulopathies due to involvement in the coagulation pathways. Then, we excluded all the patients with coagulopathy diseases.^20^

### Study design and intervention 

**Figure 1:**
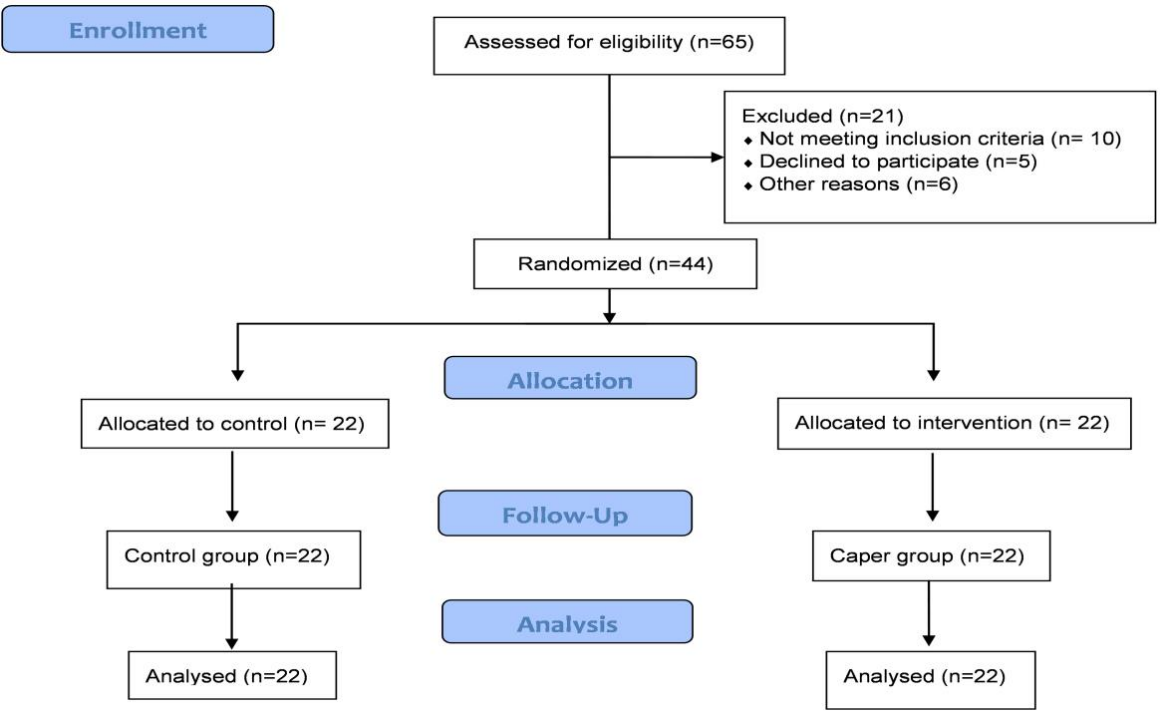
Screening, enrollment, random assignment, and follow-up of study participants

A randomized, double-blind, controlled trial was designed that aimed to assess the effects of caper fruit consumption on liver enzymes, lipid profile and grade of fatty liver in NAFLD patients. At the beginning of the study, baseline measures were recorded and qualified participants were randomly categorized by using block randomization method according to BMI. Forty four patients were randomly assigned to caper group (n=22) or control group (n=22) (Figure 1). Caper fruit was collected from Moghan, Pars Abad, Iran. The whole part of the plant was sent to the laboratory of the Herbarium Research Center, Shahid Beheshti University of Medical Sciences, Tehran, Iran (Herbarium Code: 3969). After being boiled, the fruits were soaked in home-made grape vinegar. Microbial and fungal tests were carried out before pickle distribution. The patients were advised to receive 40-50 gr of caper fruit pickles with their meals. All participants were instructed by a specialized nutritionist for lifestyle changes, similarly. Compliance of the participants was assessed by telephone interview every week. Side effects were explained to all participants and followed up each interview.

### Measures 

In the beginning, 24-hour dietary recall forms were completed and analyzed by the N4 software (Nutritionist 4, First Databank Division, Hearts Corporation). Anthropometric measures were recorded at the beginning and end of the intervention in both groups.

Blood samples of all patients were taken from the antecubital vein after 10-12 h fasting, at baseline and at 12 weeks for biochemical measurements. After centrifugation for 15 min (2500 g), the serum samples were frozen simultaneously and stored at -80 °C until analyzed. Lipid profile and liver enzyme tests were measured by an enzymatic method (Pars Azmoon Co. Kit, Tehran, Iran) using Liasys autoanalyzer.

#### Grades of fatty liver classification

Liver ultra-sonography (US) was performed by Siemens brand Sonoline G50 series - 3.5e5 MHz probe made in Germany. Liver steatosis was classified through sonographic echogenicity of liver as: 1) normal: echogenicity, the same as renal cortex; 2) grade I: mild steatosis‏ that increased liver echogenicity with visible diaphragmatic and periportal echogenicity; 3) grade II: moderate steatosis, increased liver echogenicity with imperceptible periportal echogenicity, without obscuration of diaphragm; 4) grade III: severe, steatosis; increased liver echogenicity with imperceptible periportal echogenicity and obscuration of diaphragm.^21^

#### Sample size and statistical analysis

In the present study, power of 80% with a two-sided test with α=0.05 (type I error) and mean difference of 25 IU/L for changes in ALT levels were considered to determine the sample size. On the basis of means, as stated in the previous studies,^22^ the number of participants needed to detect this difference was 22 in each group. By considering the dropout rate of 10 percent, we set the enrollment target of 25 subjects.

Numeric variables were expressed by means ± SD. The level of significance was set at P < 0.05. Statistical analyses were accomplished with IBM SPSS Statistics software (version 22; SPSS, Inc). We used Kolmogorov-Smirnov Test to assess the normal distribution of the data. Independent sample t-test was used to assess the differences in mean values of the variables between the two groups. The comparison of mean values of variables before and after the intervention in each group was examined by paired t-tests. For non-normally distributed data, appropriate non-parametric test was used. The comparison of mean change in each parameter during the trial was assessed by ANCOVA test, adjusted for baseline measures as covariates.

## Results and Discussion

Dietary intake and physical activity had no significant difference between the two studied groups (p>0.05). Baseline levels of AST, HDL.C and LDL.C were significantly different between the two groups (p<0.001). 72.7% of the participants in the caper and 59.1% of them in the control group were female. Gender distribution between the two groups were not significantly different (p=0.4). Mean age of the participants was 40.32±10.04 y in the caper and 45±10.03 y in the control group, with no significant difference between the two groups. Weight and BMI were significantly decreased in the caper (p<0.001 and p<0.001) and control groups (p=0.001 and p=0.001), respectively.

Serum ALT levels were significantly decreased at the end of the study in the caper and control groups (p<0.001 and p<0.001, respectively). Also, serum AST levels decreased in both of the studied groups (p<0.001 and p<0.001, respectively). Serum TG, TC and LDL.C were significantly decreased only in the control group (p=0.01, p<0.001 and p<0.001, respectively) (Table 1).

Adjusted to the baseline measures, the mean change in serum ALT and AST concentrations was significantly higher in the caper than the control group (p<0.001 and p=0.02, respectively). The mean change in serum of LDL.C levels was significantly higher in the control than the caper group, adjusted for the baseline measures (p=0.03) (Table 1).

At the end of the study, grade of fatty liver was significantly different between the two studied groups (p <0.001). Improve on the stage of NAFLD was significantly higher in the caper group than the control group, adjusted for the baseline measures (Table 2).

Although the standard method for NAFLD treatment is weight loss, drug and supplement trials have shown inconclusive results.^6-10^ To our knowledge, there is no concise therapy for NAFLD treatment. Thus more studies on dietary supplements and/or functional foods are needed for prevention or regression of NAFLD. Dietary supplements or functional foods which have beneficial effects on insulin resistance and blood glucose control, as well as antioxidant and anti-inflammatory activity, may be effective in the NAFLD treatment. On the other hand, many patients tend to use traditional medicine and its products for disease treatment. The present study has shown that weight loss, as the routine method for NAFLD treatment, was more pronounced in the caper than in the control L. Furthermore, a mean difference of change in serum ALT and AST concentrations was higher pronounced in the caper than the control group. Also, disease severity was significantly decreased in the caper group, adjusting for the baseline measures. Results showed that caper fruit pickle consumption in the diet can show fatty liver progression. Our results are in agreement with the previous study showing the weight-reducing effect of caper.^23^

Caper fruit, as an ITM herb, has a bitter taste which is not favored by consumers. Therefore, we prepared its pickle for patients’ convenience. Numerous studies are available assessing the effect of caper components, including fruit, seed, stem, leaf and the whole of the plant in the treatment of several other diseases such as metabolic syndrome and type II diabetes.^18,19,24^ The anti-hyperglycemic property of caper is due to the reduced absorption of carbohydrates from the small intestine, increased glucose uptake in the tissue, glucose-depletion in the liver, and the regeneration or protection of the beta cells of the pancreas.^21^ Caper is known as an herb with antioxidant properties.^16^ In addition to the effect of alcoholic and aqueous extract of caper on lipid profile, it is shown that it has hepato- and nephroprotctive effects against toxins.^24,25^ The most useful effects of caper fruit pickle on health are due to high bioactive compounds, especially polyphenols.^25,26^ According to the ITM opinion, caper has an important role in spleen performance, excretion of toxins, as well as excess “Soda” and “Balgham”, from the liver.^27^ One study reported that “Balgham”, as a component of quadruple humors, has a relationship with lipid profile.^28^

**Table 1:** Anthropometric and biochemical measurement characteristics at baseline and weeks 12 between the groups^a^

**Variables**	**Caper (n=22)**		**Control (n=22)**		**p value** ^†^
	**Baseline**	**Weeks 12**	**Before**	**After**	
**Weight (kg)**	81.32±9.93	78.93±9.95	81.32±13.92	79.77±13.21	0.08
**p value** ^‡^	<0.001		0.001		
**BMI (kg/m2)**	27.76±3.22	26.94±3.21	31.5±2.24	30.93±2.29	0.2
**p value**	<0.001		0.001		
**ALT(U/L)**	71.09±31.4	42.18±24.10	70.95±9.94	63.59±12.06	<0.001
**p value**	<0.001		<0.001		
**AST(U/L)**	38.91±13.16	27.50±10.91	67.73±10.18	59.95±14.1	0.02
**p value**	<0.001		<0.001		
**TC (mg/dl)**	191.28±47.19	183.87±44.79	166.77±37.09	152.54±35.88	0.09
**p value**	0.25		<0.001		
**TG (mg/dl)**	189.19±55.98	190.76±79.85	225±90.5	215.68±93.54	0.053
**p value**	0.9		0.01		
**LDL(mg/dl)**	112.05±35.82	103.1±280.08	77.27±44.55	64.86±40.76	0.03
**p value**	0.1		<0.001		
**HDL (mg/dl)**	38.42±8.16	39.05±7.82	44.04±5.22	44.13±6.04	0.3
**p value**	0.7		0.93		

^a^ Values are means ± SE. p < 0.05 was considered as significant BMI: body mass index; ALT: alanine transaminase; AST: aspartate transaminase; TC: total cholesterol; TG: triglycerides ^†^p values are related to the differences between the groups after 12 wk of treatment; evaluated by using an ANCOVA with baseline values as covariate ^‡^p values are related to the differences within the groups from baseline to the end; evaluated by paired sample t-test

**Table 2:** Grade of fatty liver in the groups before and after the 12-wk intervention

**Group **	**Grade of fatty liver**	**Baseline** **N (%)**	**Weeks 12** **N (%)**	**Changes** ^†^ **, N**	**p value**
Control	Normal Stage 1 Stage 2 Stage 3	0 12(54.5) 9 (40.9) 1 (4.5)	1 (4.5) 12(54.5) 8 (36.4) 1 (4.5)	Without change: 18 1 degree reduction: 4 2 degree reduction: 0	**<0.001**
Caper‏	Normal Stage 1 Stage 2 Stage 3	0 13 (59.1) 7 (31.8) 2 (9.1)	2 (9.1) 13 (59.1) 6 (27.3) 0	Without change: 15 1 degree reduction: 5 2 degree reduction: 1	

*P value by using chi-square test; ^†^Reduction in the grade of NAFLD after 12 weeks of the study

After 12 weeks of daily caper fruit consumption, ALT and AST reduction were higher compared with the control group. One study showed the same results. Streptozotocin induced diabetic rats were treated with the caper root extract for four weeks. Serum glucose level was decreased without any change in serum insulin level. Also, liver enzyme tests were significantly decreased in the caper extract-fed group.^29^ One study has shown that *Capparis spinosa* as an antidiabetic plant is capable to reduce blood glucose in streptozotocin-induced diabetic mice.^30^ In another study with the aim of evaluaaing the hepatoprotective effect of C.spinosa, ethanolic root bark extract of this medicinal plant was evaluated in a mouse model of CCl_4_-induced hepatotoxicity. Serum liver enzymes were reduced in the *C-spinosa* supplemented group with ethanolic extract of *C.spinosa.*^31^ In contrast, another study showed no significant effect of aqueous extract of caper fruit on liver enzyme tests in patients with type II diabetes.^19^ Serum liver enzymes were in normal range in the mentioned study, which may be one of probable reasons for this result. On the other hand, the effect of caper on reducing serum glucose and lipid profile is dose-dependent manner. Therefore, inconclusive results of the previous studies may be due to the effective dose. Also, caper fruit may have various components and/or amounts of polyphenols in different regions depending on the cultivation soil. Serum levels of lipid profile and/or liver enzymes shown inconsistency in various studies. These differences lead to various results.

One study assessed the effects of the caper fruit extract on the serum levels of ALT, AST, ALP, bilirubin, creatinine, urea and uric acid, as well as histo-pathologic properties of the liver, kidneys, pancreas and stomach in a rat model of type 1 diabetes.^32^ Cellular necrosis occurred in the liver, pancreas and kidneys of diabetic rats, but changes in the diabetic treated group with caper fruit extract were lower than the control group. Similarly, the serum levels of creatinine, liver enzymes, and other factors decreased. Another study assessed the anti-inflammatory properties of caper in the cell line. They showed that caper fruit inhibits cytokine gene expression, including IFNγ, IL-17 and IL-4. They concluded that the beneficial effects of caper fruit are due to saponins, flavonoids and alkaloids.^33^

To our knowledge, this is the preliminary RCT aimed to assess the effect of caper fruit pickle consumption on the hepatic lipid accumulation, serum lipid profile and liver enzyme tests in the patients with NAFLD. Caper has more benefits on lipid accumulation in the liver and grade of fatty liver. However, a study with more sample size and longer duration is needed before reaching conclusive results. There are some limitations to our results; firstly that NAFLD was diagnosed by biochemical and ultra-sonographic findings in our patients, which is not able to distinguish between simple fatty liver and NASH. Fibroscan, as a precise method for detection and staging of the liver diseases, is more expensive. Liver biopsy is the gold standard for NAFLD diagnosis, but it is an invasive and expensive method. Secondly, we did not match the patients according to their NAFLD at enrollementwe. We suggest that patients could be matched according to the grade of the fatty liver disease in the oncoming studies. In future studies, precise control of dietary intake and physical activity level is suggested. Also, caper components should be analyzed in future. Another study with the higher levels of these enzymes is needed to assess the effects of caper fruit on NAFLD progression. Insulin resistance, inflammatory and oxidative pathways should be assessed to determine the involved signaling pathways.

## Conclusion

The present study showed that caper fruit as an herbal drug may be effective in reducing the NAFLD progression because of natural compounds such as comarin and flavonoids. Thus, this fruit has medicinal properties. However, more studies are needed to obtain concise results.

## Acknowledgments

Authors are very thankful to all participated patients. Fund of the present study was provided by Vice chancellor for research, Tabriz University of Medical Sciences, Tabriz, Iran.

## Ethical Issues

The present study was approved by the Ethical Committee of Tabriz University of Medical Sciences, Tabriz, Iran (TBZMED.REC.1394.650).

## Conflict of Interest

The authors declare that there is no conflict of interest.

## References

[1] Berlanga A, Guiu-Jurado E, Porras JA, Auguet T (2014). Molecular pathways in non-alcoholic fatty liver disease. Clin Exp Gastroenterol.

[2] Angulo P (2002). Nonalcoholic fatty liver disease. N Engl J Med.

[3] Esposito E, Iacono A, Bianco G, Autore G, Cuzzocrea S, Vajro P (2009). Probiotics reduce the inflammatory response induced by a high-fat diet in the liver of young rats. J Nutr.

[4] Adams LA, Zein CO, Angulo P, Lindor KD (2004). A pilot trial of pentoxifylline in nonalcoholic steatohepatitis. Am J Gastroenterol.

[5] Harrison SA, Fincke C, Helinski D, Torgerson S, Hayashi P (2004). A pilot study of orlistat treatment in obese, non-alcoholic steatohepatitis patients. Aliment Pharmacol Ther.

[6] Belfort R, Harrison SA, Brown K, Darland C, Finch J, Hardies J (2006). A placebo-controlled trial of pioglitazone in subjects with nonalcoholic steatohepatitis. N Engl J Med.

[7] Lorvand Amiri H, Agah S, Tolouei Azar J, Hosseini S, Shidfar F, Mousavi SN (2017). Effect of daily calcitriol supplementation with and without calcium on disease regression in non-alcoholic fatty liver patients following an energy-restricted diet: Randomized, controlled, double-blind trial. Clin Nutr.

[8] Lorvand Amiri H, Agah S, Mousavi SN, Hosseini AF, Shidfar F (2016). Regression of Non-Alcoholic Fatty Liver by Vitamin D Supplement: A Double-Blind Randomized Controlled Clinical Trial. Arch Iran Med.

[9] Mousavi SN, Faghihi A, Motaghinejad M, Shiasi M, Imanparast F, Amiri HL, et al. Zinc and Selenium Co-supplementation Reduces Some Lipid Peroxidation and Angiogenesis Markers in a Rat Model of NAFLD-Fed High Fat Diet. Biol Trace Elem Res 2017. doi: 10.1007/s12011-017-1059-2 10.1007/s12011-017-1059-2 28577233

[10] Shidfar F, Jazayeri S, Mousavi SN, Malek M, Hosseini AF, Khoshpey B (2015). Does Supplementation with Royal Jelly Improve oxidative Stress and Insulin Resistance in Type 2 Diabetic Patients?. Iran J Public Health.

[11] Patel DK, Kumar R, Laloo D, Hemalatha S (2012). Diabetes mellitus: an overview on its pharmacological aspects and reported medicinal plants having antidiabetic activity. Asian Pac J Trop Biomed.

[12] Patel DK, Prasad SK, Kumar R, Hemalatha S (2012). An overview on antidiabetic medicinal plants having insulin mimetic propert. Asian Pac J Trop Biomed.

[13] Azaizeh H, Fulder S, Khalil K, Said O (2003). Ethnobotanical knowledge of local Arab practitioners in the Middle Eastern region. Fitoterapia.

[14] Jiang HE, Li X, Ferguson DK, Wang YF, Liu CJ, Li CS (2007). The discovery of Capparis spinosa L. (Capparidaceae) in the Yanghai Tombs (2800 years b.p.), NW China, and its medicinal implications. J Ethnopharmacol.

[15] Khanfar MA, Sabri SS, Zarga MH, Zeller KP (2003). The chemical constituents of Capparis spinosa of Jordanian origin. Nat Prod Res.

[16] Yang T, Liu YQ, Wang CH, Wang ZT (2008). Advances on investigation of chemical constituents, pharmacological activities and clinical applications of Capparis spinosa. Zhongguo Zhong Yao Za Zhi.

[17] Matthaus B, Ozcan M (2005). Glucosinolates and fatty acid, sterol, and tocopherol composition of seed oils from Capparis spinosa Var. spinosa and Capparis ovata Desf. Var. canescens (Coss.) Heywood. J Agric Food Chem.

[18] Eddouks M, Lemhadri A, Michel JB (2005). Hypolipidemic activity of aqueous extract of Capparis spinosa L. in normal and diabetic rats. J Ethnopharmacol.

[19] Huseini HF, Hasani-Rnjbar S, Nayebi N, Heshmat R, Sigaroodi FK, Ahvazi M (2013). Capparis spinosa L. (Caper) fruit extract in treatment of type 2 diabetic patients: A randomized double-blind placebo-controlled clinical trial. Complement Ther Med.

[20] Wang H, Wang H, Shi S, Duan J, Wang S (2012). Structural characterization of a homogalacturonan from Capparis spinosa L. fruits and anti-complement activity of its sulfated derivative. Glycoconj J.

[21] Goodman E, Daniels SR, Morrison JA, Huang B, Dolan LM (2004). Contrasting prevalence of and demographic disparities in the World Health Organization and National Cholesterol Education Program Adult Treatment Panel III definitions of metabolic syndrome among adolescents. J Pediatr.

[22] Soza A, Riquelme A, Gonzalez R, Alvarez M, Perez-ayuso RM, Glasinovic JC (2005). Increased orocecal transit time in patients with nonalcoholic fatty liver disease. Dig Dis Sci.

[23] Rahnavard R, Razavi N (2016). A review on the medical effects of Capparis spinosa L. Adv Herb Med.

[24] Tlili N, Feriani A, Saadoui E, Nasri N, Khaldi A (2017). Capparis spinosa leaves extract: Source of bioantioxidants with nephroprotective and hepatoprotective effects. Biomed Pharmacother.

[25] Nabavi SF, Maggi F, Daglia M, Habtemariam S, Rastrelli L, Nabavi SM (2016). Pharmacological Effects of Capparis spinosa L. Phytother Res.

[26] Mansour RB, Jilani IB, Bouaziz M, Gargouri B, Elloumi N, Attia H (2016). Phenolic contents and antioxidant activity of ethanolic extract of Capparis spinosa. Cytotechnology.

[27] Nazem Jahan MA. Eksir -e- Aazam [Great Elixir]: (Diseases). Tehran: Sarir -e- Ardehal; 2008. [In Persian].

[28] Emtiazy M, Keshavarz M, Khodadoost M, Kamalinejad M, Gooshahgir SA, Shahrad Bajestani H (2012). Relation between body humors and hypercholesterolemia: An Iranian traditional medicine perspective based on the teaching of Avicenna. Iran Red Crescent Med J.

[29] Kazemian M, Abad M, Haeri MR, Ebrahimi M, Heidari R (2015). Anti-diabetic effect of Capparis spinosa L. root extract in diabetic rats. Avicenna J Phytomed.

[30] Eddouks M, Lemhadri A, Hebi M, El Hidani A, Zeggwagh NA, El Bouhali B (2017). Capparis spinosa L. aqueous extract evokes antidiabetic effect in streptozotocin-induced diabetic mice. Avicenna J Phytomed.

[31] Aghel N, Rashidi I, Mombeini A (2007). Hepatoprotective Activity of Capparis spinosa Root Bark against CCl4 Induced Hepatic Damage in Mice. Iran J Pharm Res.

[32] Taghavi MM, Nazari M, Rahmani R, Sayadi AR, Hajizadeh MR, Mirzaei MR (2014). Outcome of Capparis Spinosa Fruit Extracts Treatment on Liver, Kidney, Pancreas and Stomach Tissues in Normal and Diabetic Rats. Med Chem.

[33] El Azhary K, Tahiri Jouti N, El Khachibi M, Moutia M, Tabyaoui I, El Hou A (2017). Anti-inflammatory potential of Capparis spinosa L. in vivo in mice through inhibition of cell infiltration and cytokine gene expression. BMC Complement Altern Med.

